# Some Practical Hints in Testing for Albumin in Urine

**Published:** 1893-12

**Authors:** E. C. Davis

**Affiliations:** Assistant to the Chair of Gynecology and Demonstrator of Urinalysis in Atlanta Polyclinic


### SOME PRACTICAL HINTS IN TESTING FOR ALBUMIN IN URINE.

BY E. C. DAVIS, A. B. AND M. D.,

Assistant to the Chair of Gynecology and Demonstrator of Urinalysis in Atlanta Polyclinic.

It is not the intention of the contributor of this article to endeavor to offer anything new in testing for albumin in urine, but simply to offer some suggestions as to the methods of applying the old tests which have yielded him the most satisfactory results, and to call attention to several sources of error which have crept into very common use.

We must remember that urine cannot contain more than four to five per cent. by weight of albumin, for this is the amount found in blood serum, and that the albumin found in urine is serum albumin.

Serum albumin, when precipitated, is soluble in an excess of an acid, more soluble in an excess of $H\ N\ O_3$ than $H\ Cl$ or $C_2\ H_4\ O_2$ (acetic acid).

The two most reliable tests are heat and nitric acid, not in combination, but separate and distinct. When the specimen of urine to be examined is not clear, it should always be carefully filtered through clean filter paper.

Fill a test tube about oue third fnll of clear urine and apply heat, first to its upper surface, until boiling occurs, then compare this portion which has been boiled with the lower which has not been subjected to that degree of heat. If a precipitate results then boil the whole quanty of urine. This precipitate must be one of two things, either an excess of earthy phosphates or albumin. All urine must be carefully examined before a good light before deciding there is no precipitate. Now, having a precipitate, (and at this point most errors creep in) we wish to determine what it is, if earthy phosphates, then it will readily clear up on the addition of a few drops of acetic acid, never use nitric, now drop in a few drops of the acid, and if precipitate disappears then we know it was earthy phosphates, if it is unaffected by the addition of the acid as we advised, then we know it to be albumin.

I believe the addition of the acid after the boiling is better than before, for the serum-albumin is more apt to be converted into syntonin or acid albumin which is not precipitated by boiling in an acid medium, where the urine is strongly acidulated. This is especially true of $H\ NO_3$ and offers the chief objection to its use in rendering the specimen acid. Tyson says: "not only is it true that a small quantity of albumin is dissolved by a large amount of nitric acid, but it should be remembered also that if a drop or two of nitric acid be added to a specimen of albuminous urine so as to render it distinctly acid, it may happen on boiling the urine that no precipitate will appear, although much albumin be present." This is due to the fact to which I have just directed your attention, viz: the conversion of serum-albumin into acid albumin. Nitric Acid if this be true should *never* be used to *acidulate* urine.

### NITRIC ACID OR HELLERS TEST.

Never express an opinion unless two reliable tests, at least, have been used on the specimen.

Put into a perfectly clean test tube. one-half to two-thirds of an inch of Nitric Acid C. P., then tilting the test tube, and by the aid of a pipette, carefully overlie the Nitric Acid with an equal quantity of clear urine, at the point of contact if the urine contains albumin, we will have a white line or zone, whose thickness depends upon the amount of albumin present. Do not mistake the brown or purple zone for albumin, for this is due to indican and some other coloring matters of normal urine. In some febrile disturbances, however, the coloring matter is so much increased that the albumin is colored by this when precipitated.

The mixed urates give a somewhat similar zone when in excess, but if the urine be carefully filtered when cold, or the ingredients warmed before making the test, this source of error will be largely eliminated.

This test is said to answer equally well for serum, acid, or alkali albumin.

There are other very good confirmatory tests, but for every day work in the office, these are the best.—Biblography, Exam. of Urine. By Tyson.

